# Mobile phone intervention to reduce dropout from treatment at an outpatient mental health service for older people in Nigeria

**DOI:** 10.4314/ahs.v23i4.58

**Published:** 2023-12

**Authors:** Olufisayo O Elugbadebo, Akinsola A Ojagbemi, Oye Gureje

**Affiliations:** Department of Psychiatry, University of Ibadan, Nigeria

**Keywords:** Mental healthcare, access to mental health care, mental health services

## Abstract

**Background:**

Half of older Africans drop out of treatment after a single contact with biomedical mental health services.

**Objective:**

This study examined the effect of introducing a mobile phone reminder intervention delivered by volunteering health staff to reduce dropout from an outpatient mental health service for older people in Nigeria.

**Methods:**

405 patients were studied using a quasi-experimental design: 169 who attended clinic pre-intervention (2016-2017) and 236 who attended during intervention (2018-2019). We estimated annual dropout rates, reasons for dropout and predictors of drop-out.

**Results:**

We found a trend for decreasing dropout rates during intervention (p<0.001). The most common reasons for dropout were distance to the clinic (19.5%) and unavailability of a caregiver (47.6%). Current single status (O.R =2.02, 95% C. I=1.02-3.99) and treatment without adjunctive pharmacotherapy (O. R=2.14, 95% CI; 1.07-4.26) predicted dropout.

**Conclusion:**

Mobile phone call reminders improved treatment engagement in this population. Findings are important for policy to improve access to mental healthcare in Africa.

## Introduction

Continuity of care is pivotal to the provision of effective mental health care for older people. However, due to age-related disabilities and reliance on caregivers to facilitate clinic attendance, rates of dropouts have been noted to be especially high among older service users.^1^ Other factors contributing to high rates of dropout among older Africans include cultural understanding about mental illness^2^, doubts about the efficacy of biomedical treatments,^3^ and stigmatisation of people with mental illness by biomedical health staff who may have limited mental health training.^4^,^5^ Dropout may reduce the effectiveness of treatment interventions and potentially result in worse outcome of mental health conditions in old age.

There has been a massive recent rise in access to mobile technologies globally. In 2018, there were 465 million unique mobile phone subscribers across sub-Saharan Africa^6^ and 184 million subscribers in Nigeria as at December 2019.^7^ Currently, approximately 80% of the Nigerian population own mobile phones.^8^ The likelihood is that mobile phone use will continue to rapidly increase because of reduction in phone tariffs, extension of cable grids, fall in costs of satellite network new solar-powered mobile masts, cheaper wi-fi equipment and price competition. Therefore, mobile technology is likely to be available to increasing numbers of health- care workers and service users.

Digital technology-based interventions, such as mobile telephone reminders, have been found valuable in supporting the work of health staff and in ensuring treatment engagement.^9^,^10^. Several systematic reviews and meta-analyses, conducted in Africa, have shown that mobile phone reminder improves clinic attendance rates. 11–13 Globally, telephone reminder interventions have been described in mental health services focused on children and adolescents^14^,^15^ as well as in addiction services.^16^ While a few studies have also examined the effectiveness of telephone intervention to reduce dropout from treatment among older populations receiving specialist outpatient care^17^, such exploration is yet to be conducted in Nigeria where a previous study found that approximately half of those who attended clinics dropped out of treatment after a single contact with such services.^1^

Access to specialist mental health services is limited in Nigeria, and as such, there is a pressing need to improve treatment engagement among the relatively few older people who are able to access evidence-based biomedical services. For this reason, mobile phone call reminder services were recently introduced into the outpatient mental health service for older people at the University College Hospital (UCH) Ibadan Nigeria as an intervention to improve engagement with the service. The outpatient mental health service for older people at the UCH Ibadan is located within a large healthcare facility for those who are 60 years or over (Geriatric centre). The UCH Geriatric centre is a multidisciplinary one-stop-shop model that comprises both primary and specialist care services.

This study aims to examine the impact of mobile phone call reminders on clinic attendance in the first two years of its introduction. Specifically, we sought to determine whether mobile phone call reminders reduced dropout rates as a primary outcome of intervention. Our objective is based on the hypothesis that mobile phone calls are external cues that support clinic attendance. This theory is grounded in the behavioural learning model^18^. Information from this study could inform policy that promotes the use of reminder telephone services as part of routine practice to improve treatment engagement in vulnerable populations across Nigeria and applicable in other low- and middle-income countries (LMICs).

## Methods

This was a quasi-experimental pre/post analysis of two non-overlapping participants' cohorts. One selected from among persons who received care prior to the introduction of an intervention and the other from those who presented following the introduction of the intervention.

### Participants

Participants were newly registered attendees at the outpatient mental health clinic of the UCH Geriatric Centre between January 2016 and December 2019. They all had mental health conditions that required a second consultation (follow-up appointment) after the initial consultation for assessment and diagnosis. All eligible clinic attendees agreed to participate and provided either verbal (for the pre-intervention group) or written consent (for the intervention group) to participate in the study. For participants who could not give valid consent mostly due to severe dementia, consent was taken from their spouses or adult children.

**Pre-intervention group:** Comprised persons who were newly registered and attended clinic between January 2016 and December 2017. To recruit participants for the pre-intervention group, we audited the case records of new enrolees at the outpatient mental health service of the UCH Geriatric centre for the relevant years. We included records of those who had volunteered details of their telephone contact in their case records. From the records, we identified persons in the pre-intervention group who dropped out of treatment before they were validly discharged. Such persons were next contacted by telephone to provide consent, as well as information about the reasons for dropping out of treatment.

**Intervention group:** Comprised persons who were newly registered and attended clinic between January 2018 and December 2019. They had to have access to a telephone line and provide written consent to be included in the study.

### Description of intervention

The mobile phone intervention examined in the present study comprised non-automated phone call reminders of an upcoming appointment and follow-up calls for participants who missed an appointment. The primary contact for the calls was determined during enrolment into the study. Apart from the telephone contact of the patient or primary caregiver, we obtained an additional mobile phone number of a close relative who was familiar with the patient's care as a backup mobile phone contact. Participants in the intervention group received both the reminder and follow-up calls from volunteering health staff who were either nurses or clinical psychologist. For follow up calls, participants were contacted within 48hours of their scheduled appointment by volunteering health staff. As in previous studies^19^,^20^, reminder calls were made closer (usually 24 hours) to participants' appointment. This was as we were making only one reminder call per clinic appointment. In situations where it would be difficult to take up the appointment, participants were given the chance to reschedule the appointment Only patients enrolled between January 2018 and December 2019 were followed up with reminder telephone calls in addition to the routine outpatient services rendered at the clinic.

### Outcome

The primary outcome of the present study was reduction in dropout rates.

We did not explore other secondary outcomes due to the study design, however we investigated other measures such as predictors of drop out and reasons for dropout. In line with a previous study in Nigeria, we defined dropout from treatment in patients who missed a scheduled appointment and who did not re-establish follow-up visit for at least 6 weeks after the appointment date^21^. Those who did not formally dropout were considered as regular. Patient's clinic attendance was determined from information in the case records as either dropout or regular. This was achieved through a purpose designed semi-structured questionnaire which, in addition, elicited socio-demographic information, clinical characteristics, as well as frequency and duration of scheduled appointments. The questionnaire also contained a section that could be administered to participants over the telephone.

### Statistical analysis

Descriptive statistics were used to summarize the characteristics of the participants. We estimated the dropout rate for each year and summarised the reasons given for dropout rate in the pre-intervention and intervention groups. The sociodemographic and clinical characteristics of the pre-intervention group were compared to those of the intervention group using chi squared test. Characteristics of dropout participants were also compared using the same methodology. To determine factors associated with dropout, we performed a logistic regression analysis. We first conducted an unadjusted analysis and we adjusted for factors that were significant at 5% alpha in the bivariate analysis. All statistical analyses were performed using the SPSS version 23 for Windows and level of significance was set at p<0.05.

### Ethical consideration

Ethical approval for the study was obtained from the University of Ibadan/University College Hospital Institutional Health Research Committee and informed consent was obtained from the participants.

## Results

### Participants and their baseline characteristics

**Figure 1:**
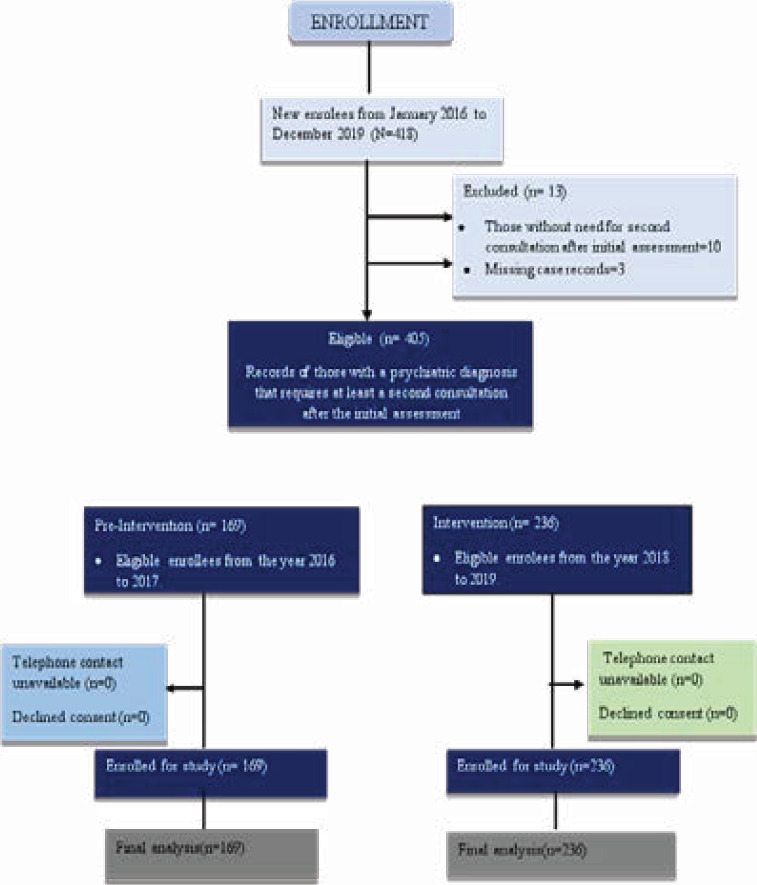
Consort Flow

A total of 405 patients were studied: 169 participants were in the pre-intervention group and 236 in the intervention group (Figure1). Table1 shows the baseline characteristics of the participants. There was a significant difference in the proportion who dropped out in the two groups: 66.9% in the pre-intervention group compared to 34.7% in the intervention group (p<0.001).

**Table 1:** Characteristics of participants, pre-intervention and intervention groups N-405

Demography	Pre-intervention	Intervention	P-value
	n (%)	n (%)	
**Age**			
60-70	56(33.1)	95(40.3)	0.115
70-79	72(42.6)	102(43.2)	
≥80	41(24.3)	39(16.5)	
**Gender**			
Male	78(46.2)	93(39.4)	0.175
Female	91(53.8)	143(60.0)	
**Educational status**			
Illiterate	29(17.2)	47(19.9)	0.539
Educated	140(82.8)	189(80.1)	
**Marital Status**			
Married	99(58.6)	111(47.0)	0.153
Unmarried	70(41.4)	125(53.0)	
			
**Living arrangement**			
Living alone	22(13.0)	11(4.7)	0.002
Living with others	147(87.0)	225(95.3)	
**Clinical Diagnosis**			
Dementia	63(37.3)	80(33.9)	0.100
Other cognitive disorders	32 (18.9)	36 (15.3)	
Mood/ Anxiety disorders	43 (25.5)	70 (29.6)	
Schizophrenia	23(13.6)	44(18.6)	
Unspecified psychiatry	8(4.7)	6(2.5)	
diagnosis			
**Clinical Attendance**			
Regular	46(27.2)	144(61.1)	<0.001
Dropout	113(66.9)	82(34.7)	
Discharged	10(5.9)	10(4.2)	
**Type of treatment**			
Psychotherapy and	180 (86.1)	173 (93.0)	0.027
Pharmacotherapy			
Psychotherapy alone	29 (13.9)	13(7.0)	

^a^ Widowed or divorce

### Dropout rate and characteristics of dropout patients

**Figure 2:**
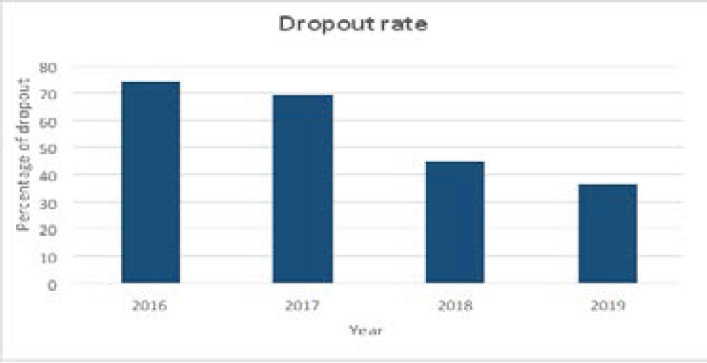
Dropout rate for each year

We obtained the dropout rate for each year after excluding those who were discharged from treatment. (See figure 2)

The two-year average dropout rates for the pre-intervention and intervention groups were 71.1% and 36.3%, respectively. Table 2 and supplementary Table 1 shows the characteristics of dropout patients in the study.

**Table 2:** Characteristics of dropout patients before and during intervention

Demography	Total	Dropout			
	N=206	Preintervention	Intervention	X^2^	P-value
		(n=113)	(n = 93)		
**Age**					
<70	71 (34.5)	36 (31.9)	35 (37.6)	0.772	0.680
70 -90	87 (42.2)	50(44.2)	37 (39.8)		
≥80	48 (23.3)	27(23.9)	21 (22.6)		
**Gender**					
Male	88 (42.7)	51 (45.1)	37 (39.8)	0.596	0.440
Female	118 (57.3)	62 (54.9)	56 (60.2)		
Educational status					
Illiterate	41 (19.9)	18 (15.9)	23 (24.7)	2.479	0.115
Educated	165 (80.1)	95 (84.1)	70 (75.3)		
**Marital status**					
Married	96 (46.6)	60 (53.1)	36 (38.7)	4.244	0.039
Not married^a^	110 (53.4)	53 (46.9)	57 (61.3)		
**Living arrangement**					
Living alone	19 (9.2)	16 (14.2)	3 (3.2)	7.284	0.007
Living with others	187 (90.8)	97 (85.8)	90 (96.8)		
**Clinical diagnosis**					
Dementia	81 (39.3)	42 (37.2)	39 (41.9)	5.798	0.326
Other cognitive	38 (18.4)	22 (19.5)	16 (17.2)		
disorders					
Mood/ anxiety	51 (24.8)	31 (27.4)	20 (21.5)		
disorders					
Schizophrenia and	31 (15)	17 (15.0)	14 (15.1)		
other psychotic					
disorder					
Unspecified psychiatry	5 (2.4)	1 (1.8)	4 (4.3)		
diagnosis					
**Type of treatment**					
Psychotherapy and	179 (86.9)	94 (83.2)	85 (91.4)	3.021	0.098
pharmacotherapy					
Psychotherapy alone	27 (13.1)	19 (16.8)	8 (8.6)		
**Comorbidity**					
Yes	120 (58.3)	71 (62.8)	49 (52.7)	2.158	0.142
No	86(41.7)	42 (37.2)	44 (47.3)		
**No of visits before dropout**					
1	98 (47.6)	54 (47.8) 59(52.2)	43 (46.2)	0.077	0.781
>1	108 (52.4)		50 (53.8)		

a Widowed or divorce

**Supplementary table 1:** Association between patient characteristics and dropout N=395

Demography	Dropout		X^2^	P-Value
	Yes (n-209)	No (n-186)		
**Age**				
<70	72 (34.4)	74 (39.8)	1.680	0.432
70 -90	91 (43.5)	79 (42.5)		
≥80	46 (22.0)	33 (17.7)		
**Gender**				
Male	92 (44.0)	73 (39.2)	0.921	0.337
Female	117 (56.0)	113 (60.8)		
**Level of Education**				
Illiterate	39 (18.7)	36 (19.4)	0.031	0.861
Education	170 (81.3)	150 (80.6)		
**Marital status**				
Married	98 (46.9)	103 (55.4)	2.836	0.092
Non-married	111 (53.1)	83 (44.6)		
**Comorbidity**				
Yes	129 (61.7)	125 (67.2)	1.288	0.256
No	80 (38.3)	61 (32.8)		
**Clinical diagnosis**				
Dementia	81 (38.8)	61 (32.8)		0.173
Other Cognitive disorders	38 (18.2)	26 (14.0)		0.225
Mood/ Anxiety disorders	51 (24.4)	61 (32.9)		0.054
Schizophrenia and other psychotic	31 (14.8)	36 (19.4)		0.064
disorder	8 (3.8)	2 (1.1)		0.084
Unspecified psychiatry diagnosis				
**Type of treatment**				
Psychotherapy and Pharmacotherapy	180 (86.1)	173 (96.0)	4.911	0.027
Psychotherapy alone	29 (13.9)	13 (7.0)		
**No of visits before dropout**				
1	96 (47.8)	44 (89.8)	28.251	0.001
>1	105 (52.2)	5 (10.2)		
**Living condition**				
Living alone	20 (9.6)	13 (7.0)	0.856	0.355
Living with others	189 (90.4)	173 (93.0)		

### Reasons for dropout

**Figure 3:**
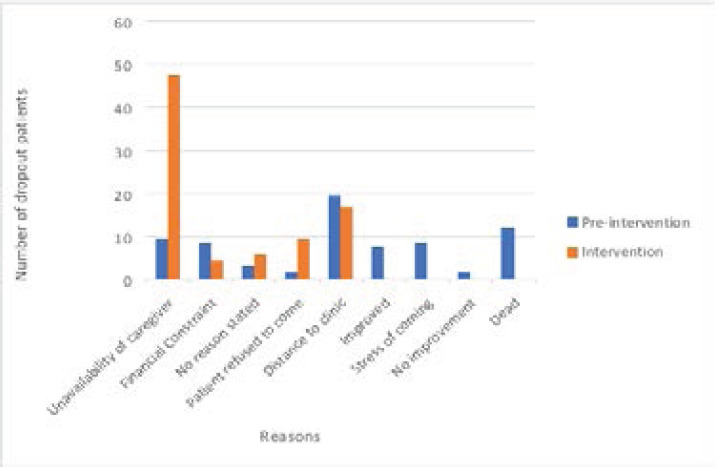
Reasons for dropout

Participants who dropped out of treatment before and during intervention were asked the reasons for their dropout. Prior to introduction of the intervention, distance from the hospital, as well as relocation to another city were the most common reasons for dropout. However, after introducing the intervention, unavailability of caregiver (75%) was the most common reason for dropout. (See figure 3).

### Predictors of dropout

In the multivariate logistic regression models adjusting for the effect of marital status, living arrangement and type of treatment received (See Table 3), being currently unmarried (either through widowhood or divorce) [Odds ratio (O.R) =2.02, 95% Confidence Interval (C.I) = 1.02-3.99)] and treatment without adjunctive pharmacotherapy (O. R=2.14, 95% CI; 1.07-4.26, p=0.031) were the predictors of dropout.

**Table 3:** Predictors of dropout

Predictors	P-Value	All	Pre-intervention	During intervention
		Odd Ratio^a^	Odd Ratio^a^	Odd Ratio
**Marital Status**				
Not married^b^	0.044	**2.02 (1.02-3.99),**	1.46 (0.67-3.20)	**1.76 (1.04 2.98)**
Married		**p=0.044**	p=0.345	**p=0.037** ^**a**^
		Reference		
				
**Living arrangement**				
Living alone	0.357	1.41 (0.68-2.92),	0.84 (0.26-2.66)	0.84 (0.24-2.94)
Living with others		p=0.357	p=0.760	p=0.779
		Reference		
				
**Type of treatment**				
Psychotherapy alone	0.031	**2.14(1.07 -4.26),**	2.16 (0.69-6.71)	1.52(0.58-3.99),
Psychotherapy and Pharmacotherapy		**p=0.031**	p=0.184	p=0.392
		Reference		

a Adjusted for Marital status, living arrangement and type of treatment

b Widowed or Divorced

## Discussion

The findings of this study show that mobile phone call reminders improved treatment engagement and led to significant reduction in dropout from an outpatient mental health service for older people in Nigeria. Dropout from the study was independently predicted by a current unmarried status and treatment without adjunctive pharmacotherapy.

Studies examining the efficacy of telephone reminder calls to improve treatment engagement among older clinic attendees are limited in the global literature. Our finding of an improved treatment engagement with the use of mobile phone reminders is similar to those reported by Dockery et al, for general practice clinics for older people in the United Kingdom.^17^ In that study, which is one of the very few to examine telephone reminders as an intervention to improve clinic attendance among older people, non-attendance rate reduced by 16% with the introduction of reminder calls. Improved clinic attendance with mobile phone call reminders have also been previously reported in general outpatient mental health services in LMICs. For example, Rajasuriyah et al found reduced non-attendance rate of 26.2% in a general outpatient psychiatric clinic in Sri Lanka.^22^ Differences between dropout rates in the present study and those of other cited reports may be due to sociocultural peculiarities of our study population as well as our protocol inclusion of the opportunity to reschedule appointments in cases where it was impracticable to visit the clinic (usually because of unavailability of a caregiver). In general, older people may be more appreciative of gestures such as phone call reminders about their health. The importance placed on official telephone contact from a healthcare provider may even be greater in sociocultural contexts in many LMICs with authoritarian orientation^23^, as well as limited access to biomedical healthcare providers.^24^

We were not surprised that unmarried status was an important correlate of dropout from treatment in the present study. In Nigeria, as it is globally, the majority of older people attending outpatient clinics come accompanied by caregivers who are mostly spouses or adult children.^25^ Where, partly due to rapid sociocultural transitions in our country context, children who are carers may not be readily available due to their own work and other personal commitments,^26^ spousal carers may be the most reliable source of support.^27^,^28^ Unmarried status in the present study was mostly due to death of or divorce from a spouse. The absence of a spouse to accompany the older patients to the outpatient mental health clinic could thus have contributed to difficulty assessing outpatient care in the present study. In line with this sequence, we found in the present study that unavailability of caregiver was the most mentioned reason for dropout. Unavailability of caregiver to accompany patient to the hospital accounted for about three-quarters of all missed appointments in the present study. Conversely, an important factor that could have contributed to the reduction in the dropout rates recorded in the intervention phase of the present study is a higher percentage of patients in this group who are living with family members.

We also found an association between dropout and type of treatment received. In this case, older persons who received basic psychotherapy, which was mostly limited to psychoeducation and counselling, without adjunctive pharmacotherapy, were more likely to dropout from treatment. This is in keeping with findings in previous studies that have reported higher rates of dropout in those receiving psychotherapy alone when compared to those on pharmacotherapy.^29^,^30^ It has been suggested that the basic tenets of conventional psychotherapeutic techniques may be discordant with some of the traditional beliefs and views about mental illness, as well as its treatment in some communities in LMICs.^31^ Such views may determine to what extent a given intervention, proffered for a mental health condition by the practitioner, is seen as credible and likely to be efficacious.^32^ Some previous studies have also suggested that many older clinic attendees in Nigeria and sub-Saharan Africa show little value for psychotherapy, and with the majority having expectations of receiving medications or injections.^24^

## Study limitations

Results of the present quasi-experimental study which was conducted in a single clinic in South-Western Nigeria are difficult to generalise in other outpatient mental health services for older people in West Africa. A multicentre study of mental health services for older people across many more countries in West Africa may provide more generalisable results. Currently, and to the best of our knowledge, the UCH Geriatric centre, where this study was conducted, is the only multidisciplinary centre dedicated to the physical and mental health care of older people in Nigeria and neighbouring West African countries. Also, we note the possibility of recall bias in the pre-intervention group, where some participants were contacted to provide information weeks after their last contact with the service. Furthermore, there is a possibility that collecting data for the two groups during different time frames may have introduced unrelated historical effects on the outcomes, however, we did not identify any significant historical event within these periods.

## Implications and impact

Findings from this study are important in designing policies aimed at promoting access to care among elderly persons in West Africa and other LMICs. First, similar to our result showing that participants were likely to drop out when they are in receipt of basic psychotherapy without adjunctive pharmacotherapy, previous studies have shown that persons requiring low intensity treatments for their mental health conditions were more likely to drop out of services provided by specialist psychiatrists.^33^ Our result thus has implication for the organization of mental health services for older people. This is so that those with conditions requiring less intense treatments are managed in primary care while specialist outpatient resources are reserved for conditions requiring more intense treatment including combined psychotherapy and pharmacotherapy. The finding in relation to dropout among our study participants who are in receipt of basic psychotherapalone may also have impact on the differences in biomedical and local cultural conceptualisation of mental illness and its treatment. Report from Nigeria and other LMICs have shown that sociocultural understanding about mental illness affects how treatment for such conditions are perceived by the people in terms of their potential efficacy.^34^ There may thus be a need to review biomedical treatment practices to align with sociocultural concepts of illness and their treatments.

Our data indicated that, unlike for the general population of clinic attendees,^33^ predetermined sociodemographic profile such as unmarried status and unavailability of a caregiver may significantly impact on dropout rates from outpatients' mental health services for older people. The policy implication of this finding may include that older people in general, but especially those with predisposing population profile for dropout from outpatient specialist services, receive community mental health services including through remote access. This study has also demonstrated that telephone calls reminder is a feasible intervention in this setting to facilitate self-regulated behaviour change of adhering to scheduled appointments which is in concordance with the behavioural learning theory, that focuses on the use of antecedents to influence behaviour. There is thus a potential for future multicentre-based studies that may explore the role of technological-based interventions in improving uptake and retention of older people in mental health services in LMICs. This could contribute to reduction in treatment gap of mental health disorders.

## Conclusion

A digital technology-based intervention comprising mobile phone call reminders was effective in reducing dropout rates in an outpatient mental health service for older people in Nigeria. However, providing basic psychotherapeutic interventions without adjunctive pharmacotherapy was a challenge to continued treatment engagement in this setting. Participants who dropped out also cited unavailability of caregivers to assist with keeping up with clinic appointments as a reason for dropout. Most of such patients had to travel long distances to attend the only service dedicated to the physical and mental health care for older people in Nigeria and surrounding West African countries. In circumstances where the few available mental health services for older people are based in geographically distant locations, community-based services may be a valuable alternative for the delivery of accessible mental health care for the large number of older people in need of mental health care in Nigeria and other similar LMICs contexts.

## References

[1] Elugbadebo O, Ojagbemi A, Adefolarin A, Gureje O (2021). Access and Discontinuity of Care at an Outpatient Mental Health Service for Older People in South Western Nigeria. Community Ment Health J.

[2] Ng T P, Nyunt M S, Z, Chiam P C, Kua E H (2011). Religion, health beliefs and the use of mental health services by the elderly. Aging & Mental Health.

[3] Okafor I, Oyewale D, Ohazurike C, Ogunyemi A (2022). Role of traditional beliefs in knowledge and perceptions of mental health and illness amongst rural-dwelling women in western Nigeria. African Journal of Primary Health Care & Family Medicine.

[4] Green J G (2020). Barriers to Mental Health Service Use and Predictors of Treatment Drop Out: Racial/Ethnic Variation in a Population-Based Study. Adm Policy Ment Health.

[5] Sirey J A (2001). Perceived Stigma as a Predictor of Treatment Discontinuation in Young and Older Out-patients with Depression. American Journal of Psychiatry.

[6] Okeleke K, Suardi S (2019). The mobile economy sub-Saharan Africa.

[7] Nigeria Communications Commission (2020). Nigeria Communications Commission Report.

[8] Silver L, Johnson C Basic mobile phones more common than smartphones in sub-Saharan Africa | Pew Research Center.

[9] Jackson K R, Booth P G, Salmon P, McGuire J (2009). The effects of telephone prompting on attendance for starting treatment and retention in treatment at a specialist alcohol clinic. Br J Clin Psychol.

[10] Rodrigues R (2012). Supporting Adherence to Antiretroviral Therapy with Mobile Phone Reminders: Results from a Cohort in South India. PLoS ONE.

[11] Griffee K, Martin R, Chory A, Vreeman R (2022). A Systematic Review of Digital Interventions to Improve ART Adherence among Youth Living with HIV in sub-Saharan Africa. AIDS Research and Treatment.

[12] Marcolino M S (2018). The Impact of mHealth Interventions: Systematic Review of Systematic Reviews. JMIR Mhealth Uhealth.

[13] Car J, Gurol-Urganci I, de Jongh T, Vodopivec-Jamsek V, Atun R, The Cochrane Collaboration (2012). Mobile phone messaging reminders for attendance at healthcare appointments. in Cochrane Database of Systematic Reviews.

[14] Sawyer S, Zalan A, Bond L (2002). Telephone reminders improve adolescent clinic attendance: A randomized controlled trial. J Paediatr Child Health.

[15] O'Brien G, Lazebnik R (1998). Telephone Call Reminders and Attendance in an Adolescent Clinic. PEDIATRICS.

[16] Johnson N A (2015). Effect of telephone follow-up on retention and balance in an alcohol intervention trial. Preventive Medicine Reports.

[17] Dockery F, Rajkumar C, Chapman C, Bulpitt C, Nicholl C (2001). The effect of reminder calls in reducing non-attendance rates at care of the elderly clinics. Postgrad Med J.

[18] Munro S, Lewin S, Swart T, Volmink J (2007). A review of health behaviour theories: how useful are these for developing interventions to promote long-term medication adherence for TB and HIV/AIDS?. BMC Public Health.

[19] Leong K C (2006). The use of text messaging to improve attendance in primary care: a randomized controlled trial. Family Practice.

[20] Corey J, Melder A (2018). Short message service (SMS) appointment reminders: A Rapid Review.

[21] Adelekan M L, Ogunlesi A O (1990). Defaulting at the Nigeria National Neuropsychiatry Hospital. Psychiatry Bulletin.

[22] Rajasuriya M, de Silva V, Hanwella R (2010). Effectiveness of reminders in reducing non-attendance among out-patients. The Psychiatrist.

[23] Chien C-L (2016). Beyond Authoritarian Personality: The Culture-Inclusive Theory of Chinese Authoritarian Orientation. Front Psychol.

[24] Ojagbemi A, Daley S (2015). Implementing the Dementia Carers Support Initiative of the National Institute for Health and Care Excellence in a sub-Saharan African Setting. Journal of Health Care for the Poor and Underserved.

[25] Uwakwe R, Modebe I (2007). Disability and care-giving in old age in a Nigerian community. Niger J Clin Pract.

[26] Jiménez S, Bueno B, Navarro A B (2022). Do the care-giving spouses of people with dementia in Spain perceive the same barriers for taking part in interventions as care-giving offspring?. Health Social Care Comm.

[27] Conde-Sala J, Garre-Olmo J, Turró-Garriga O, Vilalta-Franch J, López-Pousa S (2010). Differential features of burden between spouse and adult-child caregivers of patients with Alzheimer's disease: An exploratory comparative design. International Journal of Nursing Studies.

[28] Macleod A, Tatangelo G, McCabe M, You E (2017). There isn't an easy way of finding the help that's available. Barriers and facilitators of service use among dementia family caregivers: A qualitative study. International Psychogeriatrics.

[29] Centorrino F (2001). Factors associated with noncompliance with psychiatric outpatient visits. Psychiatr Serv.

[30] Khazaie H, Rezaie L, de Jong D M (2013). Dropping out of outpatient psychiatric treatment: a preliminary report of a 2-year follow-up of 1500 psychiatric outpatients in Kermanshah, Iran. Gen Hosp Psychiatry.

[31] Scorzelli J F, Scorzelli M R (1994). Cultural sensitivity and cognitive therapy in India. Counsel Psychol.

[32] Gureje O, Ojagbemi A (2019). Examining the “Social” in social psychiatry: The changing profile of context in the era of globalization and epidemiological transitions, with a special focus on Sub-Saharan Africa. World Social Psychiatry.

[33] Fernández D, Vigo D, Sampson NA, Hwang I, Aguilar-Gaxiola S, Al-Hamzawi AO, Alonso J, Andrade LH, Bromet EJ, de Girolamo G, de Jonge P, Florescu S, Gureje O, Hinkov H, Hu C, Karam EG, Karam G, Kawakami N, Kiejna A, Kovess-Masfety V, Medina-Mora ME, Navarro-Mateu F, Ojagbemi A, O'Neill S, Piazza M, Posada-Villa J, Rapsey C, Williams DR, Xavier M, Ziv Y, Kessler RC, Haro JM (2020). Patterns of Care and Dropout Rates from Outpatient Mental Healthcare in Low, Middle- and High-Income Countries from the World Health Organizations World Mental Health Survey Initiative. Psychological Medicine.

[34] Ojagbemi A, Gureje O, Bughra D, Moussaoui D, Craig TJ (2021). Mental health in low and middle income (LAMI) countries. Oxford Textbook of Social Psychiatry.

